# General Strategy toward Hydrophilic Single Atom Catalysts for Efficient Selective Hydrogenation

**DOI:** 10.1002/advs.202202144

**Published:** 2022-07-07

**Authors:** Yuxuan Ling, Handong Ge, Jiawen Chen, Yuqi Zhang, Yunxia Duan, Minghui Liang, Yanjun Guo, Tai‐Sing Wu, Yun‐Liang Soo, Xiong Yin, Liming Ding, Leyu Wang

**Affiliations:** ^1^ State Key Laboratory of Chemical Resource Engineering Innovation Centre for Soft Matter Science and Engineering College of Chemistry Beijing University of Chemical Technology Beijing 100029 China; ^2^ CAS Key Laboratory of Nanosystem and Hierarchical Fabrication CAS Center for Excellence in Nanoscience National Center for Nanoscience and Technology Beijing 100190 China; ^3^ National Synchrotron Radiation Research Center Hsinchu 30076 Taiwan; ^4^ Department of Physics National Tsing Hua University Hsinchu 30013 Taiwan

**Keywords:** cation‐exchange, hydrophilicity, selective hydrogenation, single atom catalysts

## Abstract

Well dispersible and stable single atom catalysts (SACs) with hydrophilic features are highly desirable for selective hydrogenation reactions in hydrophilic solvents towards important chemicals and pharmaceutical intermediates. A general strategy is reported for the fabrication of hydrophilic SACs by cation‐exchange approach. The cation‐exchange between metal ions (M = Ni, Fe, Co, Cu) and Na^+^ ions introduced in the skeleton of metal oxide (TiO_2_ or ZrO_2_) nanoshells plays the key role in forming M_1_/TiO_2_ and M_1_/ZrO_2_ SACs, which efficiently prevents the aggregation of the exchanged metal ions. The as‐obtained SACs are highly dispersible and stable in hydrophilic solvents including alcohol and water, which greatly facilitates the catalysis reaction in alcohol. The Ni_1_/TiO_2_ SACs have been successfully utilized as catalysts for the selective C=C hydrogenation of cinnamaldehyde to produce phenylpropanal with 98% conversion, over 90% selectivity, good recyclability, and a turnover frequency (TOF) of 102 h^−1^, overwhelming most reported catalysts including noble metal catalysts.

## Graphical abstract

A universal “cation‐exchange” strategy has been presented for the preparation of single‐atom catalysts (SACs) on various metal oxides with hydrophilic features. The Ni_1_/TiO_2_ SACs have demonstrated excellent catalytic performance for the selective hydrogenation of C=C in cinnamaldehyde to produce phenylpropanal in isopropanol, with high conversion and selectivity, good recyclability, and a turnover frequency (TOF) of up to 102 h^−1^.

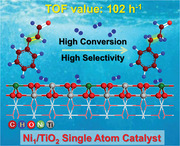

## 1 Introduction

Single atomic catalysts (SACs) have been considered as one of the significant breakthroughs in catalysis industries due to their ultrahigh atomic utilization and relatively homogeneous well‐defined active sites.^[^ ^1^, ^2^, ^3^ ^]^ Moreover, owing to the characteristics of both heterogeneous and homogeneous catalysts, SACs have exhibited remarkable enhancements in catalytic activity and exclusive selectivity.^[^ ^4^, ^5^, ^6^ ^]^ However, with the particle size reducing to the atomic scale, the surface free energy of the metals increases dramatically,^[^ ^7^ ^]^ which often leads to the aggregation of isolated atoms into nanoparticles in the synthesis process. To achieve the controllable synthesis of SACs, the ideal supports are expected to hold the merits of high specific surface area and plenty of robust anchoring sites. Additionally, the catalytic activity, selectivity, and stability of SACs would be well tuned by choosing suitable supports via changing the coordination interaction between central metal single atoms and coordination species of solid supports.^[^ ^8^, ^9^, ^10^, ^11^, ^12^, ^13^ ^]^ Thus, the controllable synthesis of SACs on suitable supports toward specific reactions has been one of the most important focuses.

Owing to the high electrical conductivity and large specific surface area of carbon supports, a considerable number of carbon‐based SACs have been developed for electrochemical applications including oxygen reduction reaction, hydrogen evolution reaction, carbon dioxide reduction reaction, etc.^[^ ^14^, ^15^, ^16^ ^]^ More importantly, the good dispersibility and hydrophilicity of SACs are prerequisites for the catalytic reaction in hydrophilic solvents such as alcohols and water.^[^ ^17^, ^18^ ^]^ Metal oxides with hydrophilic surface, adjustable composition, versatile structures, and thus huge flexibility in physicochemical properties, have been widely developed as diverse supports to fabricate SACs for different reactions.^[^ ^19^, ^20^, ^21^, ^22^ ^]^ For example, the single‐site mesoporous Pd/Al_2_O_3_ catalyst showed satisfactory selective aerobic oxidation of allylic alcohols.^[^ ^23^ ^]^ Au/FeO*_x_*, Au/CeO_2_, Pt_1_/FeO*_x_*, Pt/TiO*_x_*, Pd/La‐modified Al_2_O_3_ SACs presented both high activity and stability for CO oxidation.^[^ ^24^, ^25^, ^26^, ^27^, ^28^ ^]^ Au/CeO_2_ and Pt/CeO_2_ SACs could run the water‐gas shift reaction well.^[^ ^29^, ^30^ ^]^ To the best of our knowledge, the syntheses of aforementioned SACs are mainly based on the impregnation and coprecipitation, vacancy design, and photochemical reduction. Despite great progress in the fabrication of metal oxide‐supported SACs, it is highly desirable but challenging to precisely control single atoms on various metal oxides with good dispersibility and hydrophilicity.^[^ ^31^ ^]^

**Figure 1:**
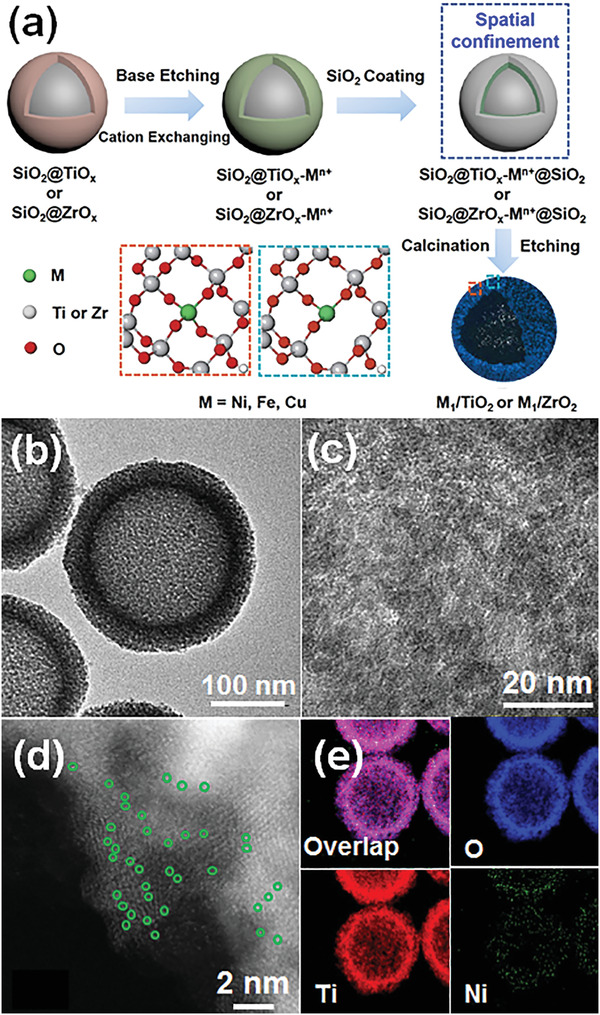
a) Synthetic route of M_1_/TiO_2_ and M_1_/ZrO_2_ SACs (M = Ni, Fe and Cu) via cation‐exchange, b) TEM image, c) HRTEM image, d) HAADF‐STEM image, and e) elemental mapping of Ni_1_/TiO_2_ SACs prepared at 800 °C. Single atomic Ni sites are highlighted by green circles in (d).

Here, we report a general “cation‐exchange” strategy to fabricate M_1_/TiO_2_ and M_1_/ZrO_2_ SACs (M = Ni, Fe, Cu) under mild conditions. In brief, a thin layer of amorphous TiO_2_ (or ZrO_2_) on the SiO_2_ nanosphere was partially etched by NaOH aqueous solution to create abundant anchoring sites for single metal atoms (**Figure** **1**a). Thereafter, the active center metal (M = Ni, Fe, Co, Cu) was introduced through “cation‐exchange” by replacing the sodium ions originating from NaOH etching. And then, the hollow nanosphere SACs were obtained through out‐layer silica coating, calcination and final silica removal by etching. The cation‐exchange and silica coating efficiently prevent the atomically dispersed metals from agglomeration during the calcination (Figure 1a). As a result, the SACs are highly dispersible in hydrophilic solvents, i.e., isopropanol, and the as‐obtained Ni_1_/TiO_2_ SACs demonstrate excellent performance in selective C=C hydrogenation of cinnamaldehyde to produce phenylpropanal with 98% conversion, over 90% selectivity and a turnover frequency (TOF) of 102 h^−1^, which are vast improvements over catalysts reported in the current literature including noble metal catalysts. Meanwhile, the Ni_1_/TiO_2_ SACs show excellent stability after multiple catalytic runs, implying good recyclability of the catalyst.

## 2 Results and Discussion

As shown in Figure S1 (Supporting Information), the surface of both SiO_2_ and SiO_2_@TiO_x_ were relatively smooth, however, it became obviously rough after NaOH etching and Ni^2+^ exchange. Further SiO_2_ coating reverted the surface of SiO_2_@TiO*_x_*‐Ni^2+^@SiO_2_ to smooth, thereby showing that the SiO_2_ coating was successful. It should be mentioned that the NaOH etching introduced many Na^+^ ions by replacing Ti atoms in TiO*_x_*. The Na^+^ ions were thereafter replaced by Ni^2+^ via the cation‐exchange step. After the Ni^2+^‐exchange, the amount of Na^+^ decreased from 0.18 to 0.10 wt%, confirmed by the inductively coupled plasma atomic emission spectroscopy (ICP‐AES) measurement. The calcination under inert atmosphere promoted the phase transformation from amorphous TiO*_x_* to anatase and the formation of Ni_1_/TiO_2_ SACs. Thereafter, with the removal of SiO_2_ by NaOH etching, hollow porous Ni_1_/TiO_2_ SACs with a ≈20 nm thick anatase shell were successfully obtained (Figure 1b). High resolution transmission electron microscopy (HRTEM) image (Figure 1c) revealed that the shell consisted of small TiO_2_ nanoparticles (NPs). According to the X‐ray diffraction patterns (XRD) (Figure S2, Supporting Information), the crystal size of anatase TiO_2_ NPs was estimated to be ≈3.5 nm by Scherrer's formula, and no Ni clusters, NiO or other titanate were observed in Ni_1_/TiO_2_ SACs. The atomically dispersed Ni atoms on TiO_2_, circled in green (Figure 1d), were further confirmed through high‐angle‐annular‐dark‐field scanning transmission electron microscopy (HAADF‐STEM). The energy dispersive X‐ray spectrum (EDS) mapping recorded with a scanning transmission electron microscope (STEM) also revealed the uniform distribution of Ni, O, and Ti elements (Figure 1e). The mass percentage of Ni species was about 0.4 wt% according to the ICP‐AES measurement. However, with the increase of Ni loading amount, several Ni nanoparticles (NPs) could be observed on the TiO_2_ nanoshells (Figure S3, Supporting Information). Without the outer silica confinement, the sample aggregated severely, and the hollow TiO_2_ sphere structure even totally collapsed during the calcination (Figures S4a,b, Supporting Information). Meanwhile, partial anatase nanoparticles transformed into the rutile phase (Figure S4c, Supporting Information), further implying that the outer silica coating is necessary for the fabrication of Ni_1_/TiO_2_ SACs.

**Figure 2:**
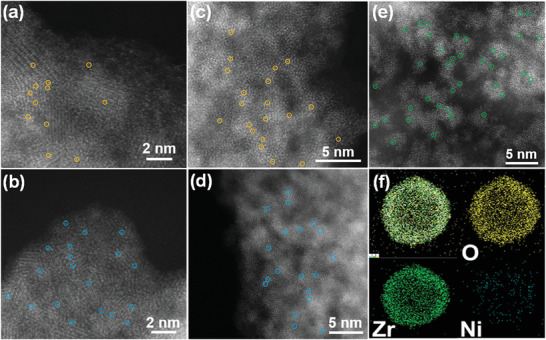
HAADF‐STEM images of SACs prepared on various oxide supports, sintered at 800 °C: a) Fe_1_/TiO_2_, b) Cu_1_/TiO_2_, c) Fe_1_/ZrO_2_, d) Cu_1_/ZrO_2_, e) Ni_1_/ZrO_2_; f) Elemental mapping image of Ni_1_/ZrO_2_ SACs. The corresponding single atomic Fe sites, Cu sites, and Ni sites are highlighted by circles.

Additionally, the traditional soaking method was applied as an alternative to the cation‐exchange process to prepare the Ni_1_/TiO_2_ SACs. In the soaking procedure, the SiO_2_@TiO*_x_* was dispersed in a Ni species contained aqueous solution to adsorb Ni^2+^ ions, the concentration of which is the same as that used in the cation‐exchange process. Unfortunately, Ni NPs could be readily observed on the TiO_2_ shells of the final products, as revealed by the transmission electron microscopy (TEM) (Figure S5a, Supporting Information) and XRD (Figure S5b, Supporting Information) characterizations, even though the outerface of the SiO_2_@TiO*_x_* nanostructures had been coated with the silica of the same thickness as before. All these results suggest that the outerface silica coating and cation‐exchange steps are necessary for the synthesis of Ni_1_/TiO_2_ SACs. It is found that the sintering temperature also affected the architecture of the final sample. For example, the Ni species on the TiO_2_ nanoshells aggregated severely upon the calcination at extra high temperatures, i.e., at 900 °C (Figure S6, Supporting Information). More importantly, this general “cation‐exchange” strategy can be easily extended to various SACs, such as Fe_1_/TiO_2_ (**Figure** **2**a), Cu_1_/TiO_2_ (Figure 2b), Fe_1_/ZrO_2_ (Figure 2c), Cu_1_/ZrO_2_ (Figure 2d), and Ni_1_/ZrO_2_ (Figure 2e,f; Figure S7, Supporting Information). The corresponding mass percentage of Fe, Cu, and Ni elements in SACs was 0.5, 0.6, 0.3, 0.5, and 0.5 wt% in Fe_1_/TiO_2_, Cu_1_/TiO_2_, Fe_1_/ZrO_2_, Cu_1_/ZrO_2_, and Ni_1_/ZrO_2_, respectively, measured by ICP‐AES and summarized in Table S1 in the Supporting Information.

**Figure 3:**
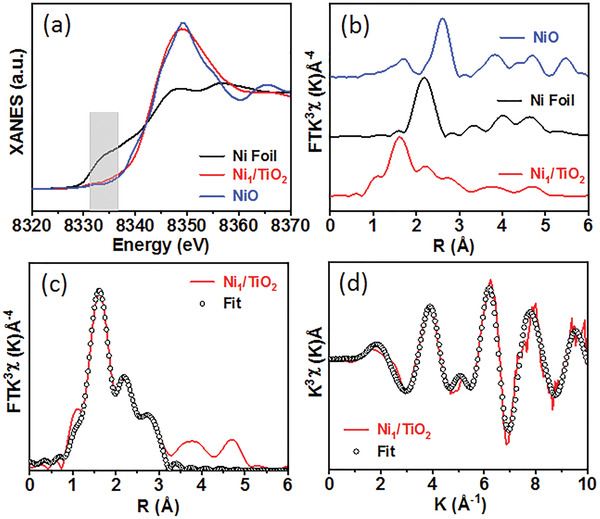
The XAS analysis of Ni_1_/TiO_2_ SAC prepared at 800 °C: a) Ni K‐edge XANES spectra; b) Ni K‐edge FT‐EXAFS spectra of Ni_1_/TiO_2_ and reference samples; c,d) EXAFS curve and the fitting curves of Ni_1_/TiO_2_ at *R* and *K* space.

To further verify the atomic dispersion of Ni in Ni_1_/TiO_2_ SACs, X‐ray absorption near‐edge structure (XANES) and extended X‐ray absorption fine structure (EXAFS) spectra were measured. As shown in **Figure** **3**a, the Ni *K*‐edge absorption of Ni/TiO_2_ SACs was higher than that in Ni foil, but slightly lower than that in NiO, indicating that the valence state of Ni species in the Ni_1_/TiO_2_ SACs was close to +2 as that in NiO. The Fourier‐transformed (FT)^2^‐weighted EXAFS spectrum of Ni_1_/TiO_2_ (Figure 3b; Table S2, Supporting Information) revealed a primary peak at 2.04 Å assigned to Ni–O coordination, and the lack of Ni–Ni characteristic peak (2.52 Å) also confirmed the atomic dispersion of single Ni atoms, which are consistent with the characterization results of AC HAADF‐STEM and XRD. Based on the fitting results of EXAFS of the Ni_1_/TiO_2_ SACs (Figure 3c,d), the average coordination number (CN) of the isolated Ni atom in the Ni_1_/TiO_2_ SACs was 3.5 ± 0.9 in the form of Ni–O (Table S2, Supporting Information). Moreover, the XANES and EXAFS results of the Cu atoms dispersed on the TiO_2_ shell confirmed the atomic dispersion of Cu species within the Cu_1_/TiO_2_ SACs (Figure S8 and Table S2, Supporting Information). In addition, Cu atoms were atomically dispersed within the ZrO_2_ shell in Cu_1_/ZrO_2_ SAC sample, verified by its XANES and EXAFS measurements (Figure S9 and Table S3, Supporting Information).

**Figure 4:**
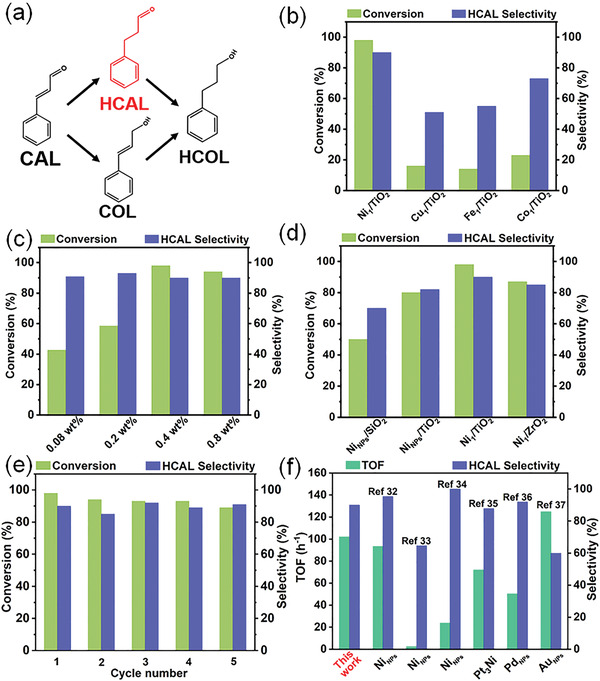
a) Scheme of the reaction path for the selective hydrogenation of cinnamaldehyde; b) catalytic performance of different SACs toward the selective hydrogenation of cinnamaldehyde; c) catalytic performance of Ni_1_/TiO_2_ SACs with different Ni loading amounts; d) catalytic performance of different catalysts, including Ni_NPs_/SiO_2_, Ni_NPs_/TiO_2_, Ni_1_/TiO_2_, and Ni_1_/ZrO_2_; e) recyclability test for Ni_1_/TiO_2_ SACs toward the selective hydrogenation of cinnamaldehyde; f) performance comparison (TOF and selectivity) of different heterogeneous catalysts for the selective hydrogenation of cinnamaldehyde.

It is well known that both C=C hydrogenation product (phenylpropanal, HCAL) and C=O hydrogenation product (cinnamyl alcohol, COL) of cinnamaldehyde (CAL) are important chemical and pharmaceutical intermediates (**Figure** **4**a).^[^ ^32^, ^33^, ^34^, ^35^, ^36^, ^37^, ^38^, ^39^, ^40^ ^]^ Consequently, the Ni_1_/TiO_2_ SACs, with a Brunauer–Emmett–Teller (BET) surface area of up to 284 m^2^ g^−1^ (Figure S10, Supporting Information), were utilized for the selective hydrogenation of C=C in cinnamaldehyde under optimal conditions. Moreover, its Zeta potential in aqueous solution was −34 mV, indicating its highly hydrophilic nature,^[^ ^41^, ^42^ ^]^ which is further confirmed by the ultrasmall contact angle that is almost undetectable. Taking high conversion (98%) together with excellent selectivity (90%) into consideration, the reaction temperature, H_2_ pressure and the calcination temperature of the SACs were optimized to 130 °C (Figure S11 and Table S4, Supporting Information), 3 MPa (Figure S12 and Table S5, Supporting Information), and 800 °C (Figure S13 and Table S6, Supporting Information), respectively. To investigate the metal‐dependent catalysis, four TiO_2_‐supported SACs, including Fe_1_/TiO_2_, Co_1_/TiO_2_, Ni_1_/TiO_2_, and Cu_1_/TiO_2_ SACs were adopted for the hydrogenation of cinnamaldehyde (Figure 4b; Table S7, Supporting Information). All catalysts presented high selectivity towards C=C hydrogenation to produce HCAL, and the Ni_1_/TiO_2_ SACs demonstrated the highest conversion (98%) and the best selectivity (90%).

We also investigated the influence of Ni loading amount on the selective catalysis. As shown in Table S8 (Supporting Information), when no Ni was loaded onto the TiO_2_ substrates, the conversion was only 27%. With the increase of Ni loading, the conversion first increased step by step until the maximum was reached, and then dropped slightly. The highest conversion of 98% with a satisfactory selectivity of 90% to HCAL was obtained at the Ni loading amount of 0.4 wt%, (Figure 4c; Table S8, Supporting Information). The satisfactory catalytic performance of the Ni_1_/TiO_2_ SAC may result from the hydrogen spillover effects within the SAC catalyst (detailed in the Supporting Information), as revealed by the hydrogen temperature‐programmed desorption (H_2_‐TPD) measurement (Figure S14, Supporting Information).^[^ ^43^, ^44^, ^45^ ^]^ As aforementioned, excessive Ni loading would result in the formation of Ni nanoparticles on the TiO_2_ support (Figure S3, Supporting Information), which adversely affected the catalytic activity. As depicted in Figure 4d and Table S9 (Supporting Information), in comparison with Ni NPs supported on TiO_2_ (Ni_NPs_/TiO_2_), the Ni_1_/TiO_2_ SACs hold significant superiority in both conversion and selectivity to HCAL. Also, as for the catalytic performance, the Ni_1_/ZrO_2_ SACs significantly surpassed the Ni_NPs_/ZrO_2_ catalysts (Table S9, Supporting Information). More interestingly, the as‐prepared Ni_1_/TiO_2_ SACs demonstrated a satisfactory stability, after 5 cycles, no obvious decay was observed in the conversion and selectivity (Figure 4e; Table S10, Supporting Information). If the oxide support was changed to ZrO_2_ (Ni_1_/ZrO_2_ SACs), the catalytic performance decreased slightly (Figure 4d; Table S11, Supporting Information), suggesting that the hydrogenation reaction was also support‐dependent. The shift of binding energy of Zr 3d_5/2_ was remarkably larger than that of Ti 2p_3/2_ after being treated under H_2_ atmosphere. The results may lead to the difference in catalytic performance between Ni_1_/TiO_2_ and Ni_1_/ZrO_2_ SACs, reflected by the ex situ XPS measurements (Figure S15, Supporting Information) (detailed in the Supporting Information). In addition, the Ni_1_/ZrO_2_ showed the highest catalytic performance among the four Fe_1_/ZrO_2_, Co_1_/ZrO_2_, Ni_1_/ZrO_2_, and Cu_1_/ZrO_2_ SACs (Figure S16 and Table S11, Supporting Information). Moreover, it should be noted that, for the Ni_1_/TiO_2_ SACs (Figure S13 and Table S6, Supporting Information), the calcination temperature also influenced the catalytic activity toward the selective hydrogenation of cinnamaldehyde, and the optimized calcination temperature was 800 °C (Table S12, Supporting Information). The change in catalytic activity could be related to the decrease in BET surface area. The BET surface area of the Ni_1_/TiO_2_ sample prepared at 900 °C was ≈192 m^2^ g^−1^ (Figure S17a, Supporting Information), and the sintering temperature can influence slightly the pore structure of the final SAC (Figure S17b, Supporting Information). Moreover, when the catalyst was sintered at 900 °C, Ni NPs were also observed, as revealed by the STEM image (Figure S6, Supporting Information). These changes may lead to the decrease in catalytic activity toward the selective hydrogenation of cinnamaldehyde. When these results are compared with the previously reported catalysts (Figure 4f; Table S13, Supporting Information), including some noble metals, Ni nanoparticles (NPs) and alloyed NPs, the as‐prepared oxide supported SACs in this study showed a high TOF value of 102 h^−1^, high catalysis activity and good stability, demonstrating the SACs may be suitable for various potential catalysis applications.

## 3 Conclusion

In conclusion, we presented a general strategy to prepare hydrophilic single atom catalysts on various metal oxide‐based supports via a cation‐exchange process. The as‐obtained Ni_1_/TiO_2_ SACs showed high activity and stability toward the selective C=C hydrogenation of cinnamaldehyde. A conversion of up to 98% with a selectivity of up to 90% has been achieved in producing phenylpropanal from cinnamaldehyde. More importantly, no obvious decay in the catalytic performance (conversion efficiency and selectivity to HCAL) was observed after five cycles of reaction. This work paves a new way for the rational design of hydrophilic single atom catalysts aiming at the catalysis applications of the selective hydrogenation in hydrophilic solvents.

## 4 Experimental Section

### Materials

Titanium (IV) *n*‐butoxide (TBOT) was purchased from J&K. Tetraethyl orthosilicate (TEOS) was purchased from Alfa Aesar. Ni(NO_3_)_2_·6H_2_O, Co(NO_3_)_2_·6H_2_O, Cu(NO_3_)_2_·6H_2_O, Fe(NO_3_)_3_·6H_2_O were purchased from Xilong Chemical company. Polyvinylpyrrolidone (PVP) (*M*_w_ = 55 000), hydroxypropyl cellulose, anhydrous acetonitrile, anhydrous ethyl alcohol were purchased from Sigma Aldrich. Ammonia solution (28 wt%) was purchased from Aladdin. All reagents were used as received without further purification.

### Synthesis of M_1_/TiO_2_ and M_1_/ZrO_2_ Catalysts

Synthesis of Silica Nanoparticles—23 mL of ethanol was added into a 50 mL flask, and then 0.86 mL of TEOS was injected into ethanol. After stirring for 3 min, 4 mL of H_2_O was added, and kept stirring for another 3 min. Finally, ammonia (0.8 mL) was injected into the flask. Four hours later, the products were centrifuged, and washed with ethanol. The final samples were dispersed in 10 mL ethanol. The size of silica nanoparticles can be adjusted by changing the ratio of water and ammonia solution.

Synthesis of SiO_2_@TiO*_x_*—First, 10 mL of ethanol and 7 mL of acetonitrile were added into a flask, and stirred for 3 min. Then, 50 mg of hydroxypropyl cellulose was added to the solution, and stirred for 30 min. After the hydroxypropyl cellulose was completely dissolved, the as‐synthesized SiO_2_ spheres were injected into the solution. After stirring for 10 min, 0.2 mL of ammonia was injected. 10 min later, a TBOT buffer solution (1 mL TBOT in 3 mL ethanol and 1 mL acetonitrile) was added into the SiO_2_ solution. After 2 h of reaction, the final product (SiO_2_@TiO*_x_*) was collected by centrifugation method.

Synthesis of SiO_2_@ZrO*_x_*—First, ethanol (20 mL), H_2_O (0.1 mL), and hydroxypropyl cellulose (10 mg) were added into a flask (50 mL), and stirred for at least 15 min. After the hydroxypropyl cellulose was completely dissolved in the solution, the as‐prepared SiO_2_ was added to the above solution. After stirring for 10 min, a desired amount of ZBOT in ethanol (4.5 mL) was injected into the solution using a syringe pump at a rate of 0.25 mL min^−1^. After that, the mixture was stirred for 20 h at room temperature. Finally, the product was collected by centrifugation and washed with ethanol three times.

Metal Ions‐Exchanging Process—The as‐obtained SiO_2_@TiO*_x_* sample was treated with NaOH (0.025 mol, 10 µL 2.5 m). During the base treatment process, the NaOH can break the Ti–O bonds of amorphous titanium dioxide in SiO_2_@TiO*_x_*. The 500 µL of 0.05 mol L^−1^ Ni^2+^ solution was prepared and used for ion exchanging for 12 h, and then, the product (SiO_2_@TiO*_x_*‐Ni^2+^) was centrifuged and washed with deionized water for 3 times. The loading amount of Ni^2+^ ions could be easily adjusted by changing the concentrations of Ni^2+^ ions. In the cases of other metal ions, such as Co^2+^, Fe^3+^, and Cu^2+^, the same molar concentration of the solution was used during the ion‐exchanging step.

Synthesis of SiO_2_@TiO*_x_*‐Ni^2+^@SiO_2_–The above‐mentioned SiO_2_@TiO*_x_*‐Ni^2+^ sample was dispersed in deionized water (20 mL), and PVP (200 mg, *M*_w_ = 40 000) was added into water and stirred for 12 h to ensure that PVP was fully adsorbed on the surface of TiO_2_ particles. The product was collected by centrifugation and washed with deionized water. After that, the sample was dispersed in 23 mL of ethanol, and 4.3 mL of deionized water was added immediately. TEOS (0.86 mL) was added 3 min later. After stirring for 3 min, ammonia (0.8 mL) was added to the solution. Finally, the sample was collected by centrifugation and washed with alcohol three times.

Calcination Process—Grind the dried sample into powder first, and put it in a porcelain boat. The SiO_2_@TiO*_x_*‐Ni^2+^@SiO_2_ sample was then sintered under N_2_ atmosphere in a tube furnace. The heating rate was set to be 5 °C min^−1^, and sintered at 800 °C for 2 h. The calcination temperature could be changed to 600, 700, and 900 °C, accordingly.

Fully Etching Step—The as‐sintered SiO_2_@Ni/TiO_2_@SiO_2_ sample was dispersed in deionized water (26 mL), and then NaOH (2.5 mol L^−1^, 4 mL) solution was added into the solution. The fully etching of SiO_2_ was conducted at 90 °C in an oil bath, and reacted for 4 h to completely remove the SiO_2_ template. The final product was isolated by centrifugation and washed with deionized water to remove the excess NaOH. The sample was then transferred into an oven and dried at 60 °C, and then, the Ni_1_/TiO_2_ SA catalyst was obtained.

Synthesis of Ni_NPs_/SiO_2_—A certain quantity of commercial SiO_2_ powder was used as the matrix. The SiO_2_ was mixed with nickel nitrate solution (0.05 mmol mL^−1^) through a microfluidic pump at a rate of 0.5 mL min^−1^. They were stirred at 80 °C for 30 min. Finally, the solution was completely vaporized. The SiO_2_ powder with adsorbed Ni^2+^ ions was further dried in an oven at 60 °C overnight. Finally, the sample was transferred into a tube furnace and sintered at 500 °C for 2 h in the flow of H_2_ to obtain the Ni_NPs_/SiO_2_ sample.

Synthesis of Ni_NPs_/TiO_2_—The synthesis of Ni_NPs_/TiO_2_ was similar to that of Ni_1_/TiO_2_ SA catalyst. The only difference was that a solution with 10 times the concentration of Ni^2+^ ions (0.5 mol L^−1^) was used during the ion‐exchanging process.

Synthesis of M_1_/ZrO_2_ Catalysts—The as‐prepared SiO_2_@ZrO*_x_* sample was then used in the metal ion‐exchanging step. Fe^3+^, Co^2+^, and Ni^2+^ ions were used, respectively, to prepare different M_1_/ZrO_2_ catalysts with a recipe similar to that for M_1_/TiO_2_ sample.

### Characterization

TEM and HRTEM characterizations were performed with a JEOL JEM‐1200 microscope operated at 100 kV or a JEOL JEM‐2100F microscope operated at 200 kV. AC HAADF‐STEM images and EDS mapping profiles were recorded with a Titan Cubed Themis G2 300 microscope operated at 300 kV. The XRD patterns were measured on a Bruker AXS D8 Advance X‐ray diffractometer. The X‐ray photoelectron spectra (XPS) were collected on an ESCALAB 250 X‐ray photoelectron spectrometer, and the binding energy was corrected by C 1s (284.8 eV). The XANES and EXAFS spectra of the Cu, Ni k‐edge were measured on beamline 1W1B of the Beijing Synchrotron Radiation Source, Institute of High Energy Physics, Chinese Academy of Sciences. Ni foil, NiPc, NiO, Cu foil, CuO, and Cu_2_O were used as reference samples. The catalysts were measured in fluorescence mode. The obtained XAFS data was processed in Athena (version 0.9.26) for background, pre‐edge line and post‐edge line calibrations. Then Fourier transformed fitting was carried out in Artemis (version 0.9.26). The *k* ^3^ weighting, *k*‐range of 3–12 Å^−1^ and *R* range of 1–3 Å were used for the fitting of foils; *k*‐range of 2–11 Å^−1^ and *R* range of 1–3.2 Å were used for the fitting of other samples. The four parameters, coordination number, bond length, Debye–Waller factor and *E*_0_ shift (CN, *R*, *σ* ^2^, Δ*E*_0_) were fitted without anyone fixed, constrained, or correlated. In addition, *K*‐edge absorption near‐edge structure (XANES) and extended X‐ray absorption fine structure (EXAFS) of the Cu_1_/ZrO_2_ SAC sample was measured at the TLS17C of Taiwan Light Source, NSRRC. The software packages IFEFFIT were used to analyze this XANES and EXAFS spectra. The surface area and pore size distribution of the catalysts were obtained on Quanta chrome Instrument chemisorption Apparatus. Typically, the specific surface area was measured by the BET method, and the pore size distributions were calculated using the Barrett–Joyner–Halenda (BJH) method according to the adsorption–desorption curve. The load amount of the metals on the catalysts was determined by an iCAP 6000 series ICP‐AES. In the case of H_2_‐TPD measurement, the samples were treated at 120 °C under 10% H_2_/Ar for 2 h, and then cooled to room temperature. The samples were then heated to 900 °C at a rate of 10 °C min^−1^ after they were treated under Ar atmosphere for 30 min. The ex situ XPS spectra of the samples were collected on an ULVAC PHI5000 VersaProbe III X‐ray photoelectron spectrometer, and a transfer vessel was used to transfer the treated sample from the autoclave to the measurement chamber, preventing the sample from the exposure to the air. The samples were loaded on a stub, and then they were placed into the stainless autoclave to carry out the hydrogen reduction treatment. After treated at 130 °C for 2 h, the samples were transferred into the transfer vessel in the glove box with the oxygen content being lower than 0.1 ppm. Finally, the samples were further transferred from the vessel to the chamber to conduct the measurements.

The turnover frequency (TOF) value of the catalyst was calculated at 7% conversion (reaction time: 10 min) of the substrate (54.8 mg) and based on the total Ni species loading in the Ni_1_/TiO_2_ SAC catalyst according to Equation (1)

(1) TOF=FC/tWM

where *F* (mol) is the mole of the reactant, *C* is the conversion of the reactant, *t* (h) is the reaction time, *W* (g) is the catalyst mass, *M* (mol g^−1^) is the mole of the active Ni atom per unit mass.

### Catalytic Activity Evaluation

Cinnamaldehyde hydrogenation was performed in isopropyl alcohol in a SLM‐100 high pressure reactor (Beijing Century Langsen Company). In brief, 25.0 mg of catalyst, 50 µL (0.4 mmol) of reactant (Cinnamaldehyde) and 25.0 mL of isopropyl alcohol were sequentially added to the high pressure reactor (100 mL). The different catalysts were utilized in this reaction which was conducted under various conditions, including different H_2_ pressures and reaction temperatures. The products of hydrogenation reaction were analyzed using a GC‐2060 gas chromatograph produced by Tengzhou Lunan Instrument Analysis Company. The conversion efficiency and selectivity of catalysts were obtained by using the internal standard method with biphenyl serving as the internal standard.

## Conflict of Interest

The authors declare no conflict of interest.

## Supporting information

Supporting Information

## Data Availability

The data that support the findings of this study are available in the supplementary material of this article.
