# Supplementary material for: General Strategy toward Hydrophilic Single Atom Catalysts for Efficient Selective Hydrogenation
Source: Adv Sci (Weinh). 2022 Jul 7;9(25):2202144. doi: 10.1002/advs.202202144 (PMC9443439; doi:10.1002/advs.202202144)
Supplement: Supplementary file 1 — Supporting Information [file ADVS-9-2202144-s001.pdf]

Supporting Information

General Strategy toward Hydrophilic Single Atom Catalysts for Efficient Selective Hydrogenation

Yuxuan Ling, [a] Handong Ge, [a] Jiawen Chen, [a] Yuqi Zhang, [b] Yunxia Duan,[a] Minghui Liang, [b] Yanjun Guo, [b] Tai-Sing Wu, [c] Yun-Liang Soo, [d] Xiong Yin,[a] Liming Ding [b] and Leyu Wang*[a]*

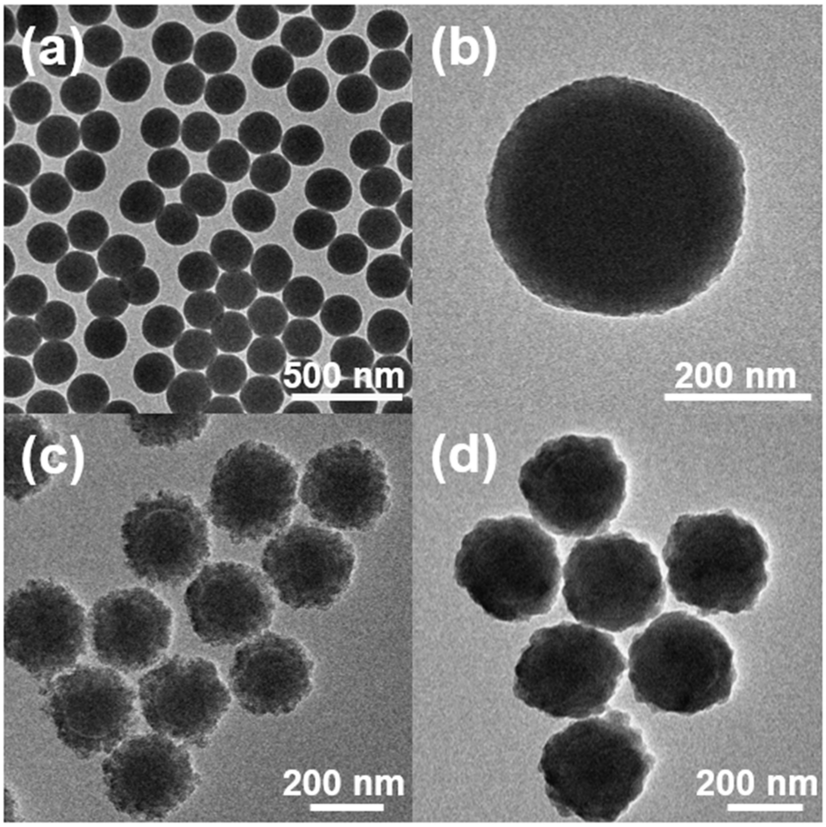

Figure S1. TEM images of (a) SiO_2, (b) SiO_2@TiO_x, (c) SiO_2@TiO_x-Ni^{2+}, and (d) SiO_2@TiO_x-Ni^{2+}@SiO_2 nanostructures.

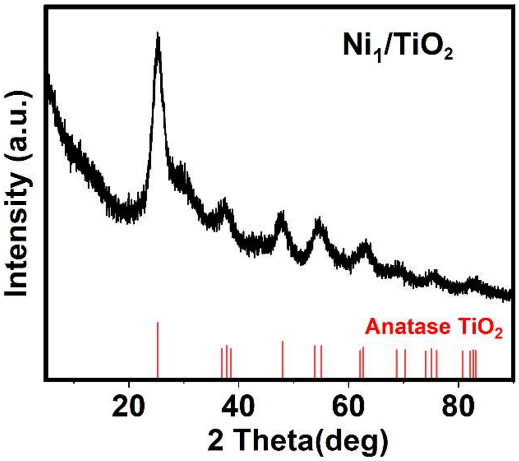

Figure S2. XRD pattern of the as-prepared Ni_1/TiO_2 SACs, sintered at 800 ºC.

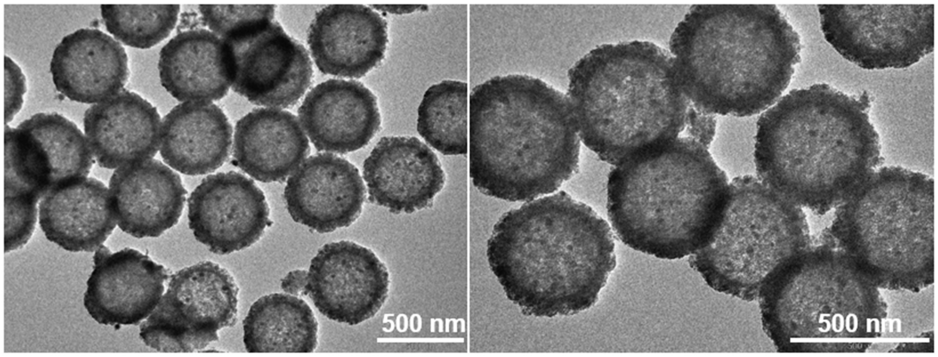

Figure S3. The typical TEM images of Ni_{NPs}/TiO_2 sample prepared at 800 ºC with high amount of Ni species via cation-exchange approach.

This result suggests that the control of Ni loading amount is critical for the formation of single atom catalysts on the surface of TiO_2 nanoshells.

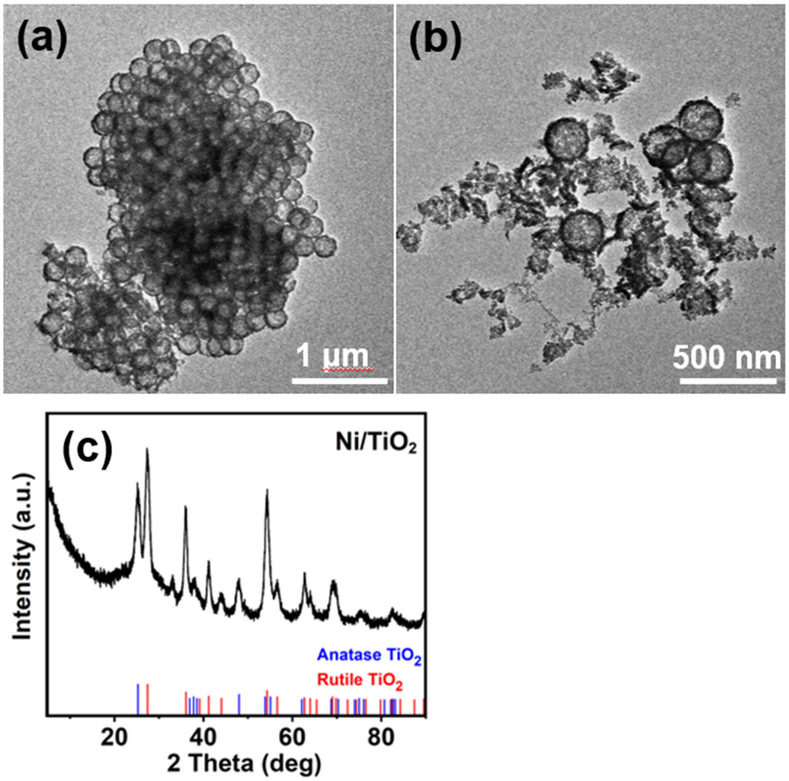

Figure S4. (a, b) TEM images and (c) the corresponding XRD patterns of the samples which were prepared by calcining the SiO_2@TiO_2-Ni^{2+} nanostructures without the protective outer layer of SiO_2.

It is clear the outer layer SiO_2 coating is necessary to maintain the hollow sphere structure of the Ni_1/TiO_2 SACs and the anatase crystal structure of TiO_2 nanoshell.

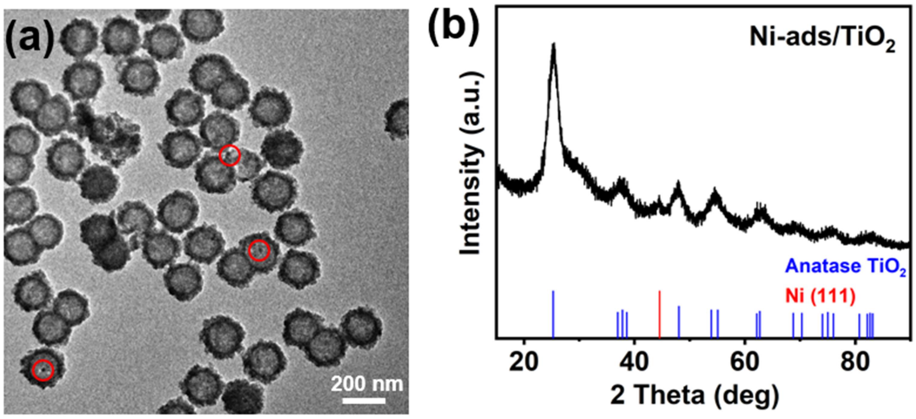

Figure S5. TEM image (a) and XRD pattern (b) of Ni-$_{ads}$/TiO_2 catalyst prepared through the traditional soaking-adsorption-calcination method.

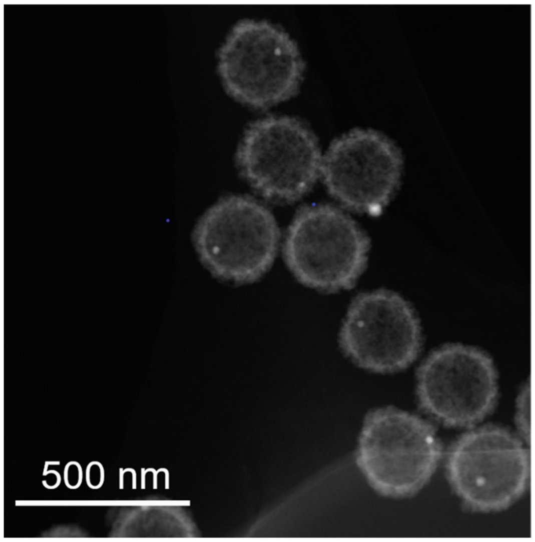

Figure S6. Typical STEM image of the Ni_1/TiO_2 SACs sintered at 900 °C.

Moreover, the sintering temperature also affected the architecture of the final sample. For example, the Ni species on the TiO_2 nanoshells may aggregate severely upon the calcination at temperatures higher than the optimized one, such as 900 ºC.

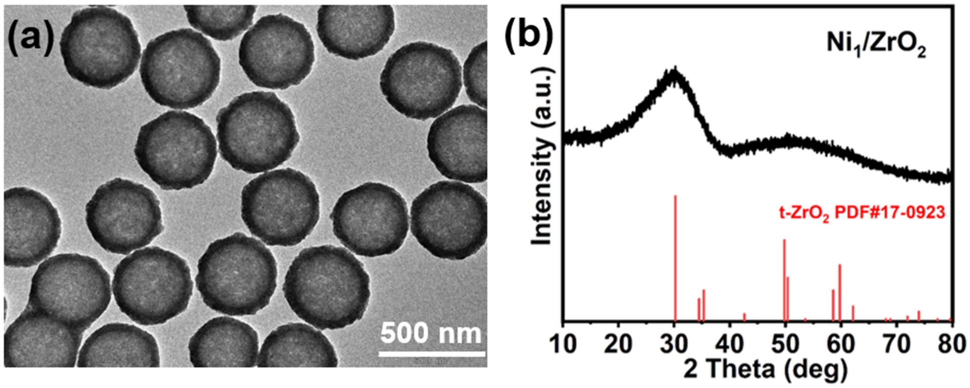

Figure S7. TEM image and XRD pattern of Ni_1/ZrO_2 SACs prepared at 800 ºC.

Table S1. Mass percentage of elements (M) in various M_1/TiO_2 and M_1/ZrO_2 SACs determined by ICP-AES.

Catalysts	Fe (wt.%)	Ni (wt.%)	Cu (wt.%)
Fe_1/TiO_2	0.5	-	-
Ni_1/TiO_2	-	0.4	-
Cu_1/TiO_2	-	-	0.6
Fe_1/ZrO_2	0.3	-	-
Ni_1/ZrO_2	-	0.5	-
Cu_1/ZrO_2	-	-	0.5

Table S2. EXAFS fitting parameters at the Cu and Ni K-edge (S_0^2= 0.87 and 0.83) in TiO_2 support-based SAC samples.

Sample	Path	C.N. [a]	R (Å) [b]	$\sigma^2 \times 10^3$ (Å^2) [c]	ΔE (eV) [d]	R factor
Ni foil	Ni-Ni	12*	2.48±0.01	6.2±0.2	7.3±0.3	0.001
Ni	Ni-O	3.5±0.9	2.04±0.02	4.7±2.8	-1.6±2.2	0.018
	Ni-Ni	1.1±0.9	2.52±0.04	6.4±10.6	2.0±6.1	
	Ni-Ti	1.8±1.5	3.12±0.06	10.0±7.3		
Cu foil	Cu-Cu	12*	2.54±0.01	8.5±0.4	4.2±0.6	0.002
Cu	Cu-O	3.2±0.4	1.95±0.01	3.3±0.1	-1.3±1.5	0.015
	Cu-Ti	1.0±0.6	3.02±0.04	7.3±5.5	-2.1±5.1	

[a]*C.N*: coordination numbers; [b]*R*: bond distance; [c]σ^2: Debye-Waller factors; [d] Δ*E*: the inner potential correction. *R* factor: goodness of fit. * the experimental EXAFS fit of metal foil by fixing CN as the known crystallographic value.

Table S3. EXAFS fitting parameters at the Cu K-edge (S_0^2=0.83) in ZrO_2 support-based SAC sample

Sample	Path	C.N. [a]	R (Å) [b]	$\sigma^2 \times 10^3$ (Å^2) [c]	ΔE (eV) [d]	R factor
Cu_1/ZrO_2	Cu-O	3.7 ± 0.3	1.94 ± 0.01	5.0 ± 1.1	0.2 ± 1.2	0.0023
	Cu-Zr	2.2 ± 0.6	3.25 ± 0.02	12.7 ± 2.5	6.9 ± 2.0	

[a]*C.N*: coordination numbers; [b]*R*: bond distance; [c]σ^2: Debye-Waller factors; [d] Δ*E*: the inner potential correction.

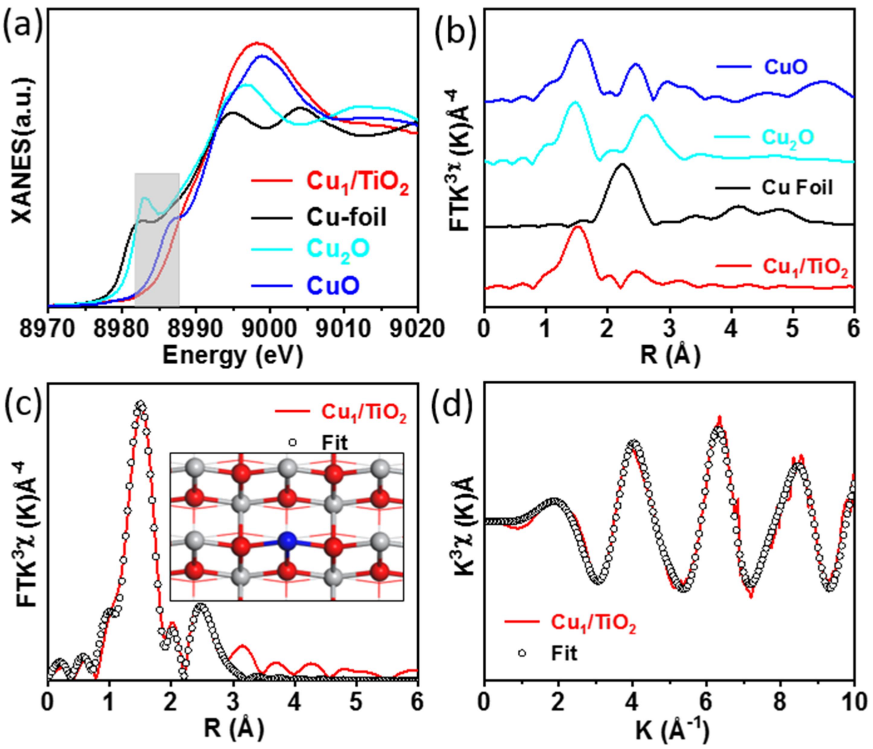

Figure S8. The XAS analysis of the Cu_1/TiO_2 SAC prepared at 800 ºC: (a) Cu K-edge XANES spectra; (b) Cu K-edge FT-EXAFS spectra of Cu_1/TiO_2 and reference samples; (c, d) EXAFS curve and the fitting curves of Cu_1/TiO_2 at R and K space.

The position of the Cu K-edge absorption threshold was the reflection of the average oxidation state of Cu species in the TiO_2 matrix. As shown in **Fig. S8a**, the edge position of Cu_1/TiO_2 was relatively close to that of CuO, which indicates that the valence state of the Cu species in the SACs was about +2. The corresponding Fourier transform (FT) EXAFS spectra of Cu_1/TiO_2 and the references (Cu foil, CuO and Cu_2O) were illustrated in **Fig. S8b**. The Cu_1/TiO_2 SACs exhibited an obvious FT peak located at ~1.95 Å, which could be mainly attributed to the scattering of Cu-O coordination. Moreover, there is no Cu-Cu shell peak at ~2.54 Å distinguished in the sample, further suggesting that only atomically dispersed Cu atoms were present in the sample. Additionally, two fitting curves matched quite well with the experiment spectra (**Figs. S8c-d**). It revealed that the Cu−O coordination number in the Cu_1/TiO_2 is 3.2 ± 0.4, with typical moieties shown in the inset of **Fig. S8c**, along with the fitting parameters summarized in **Table S2**.

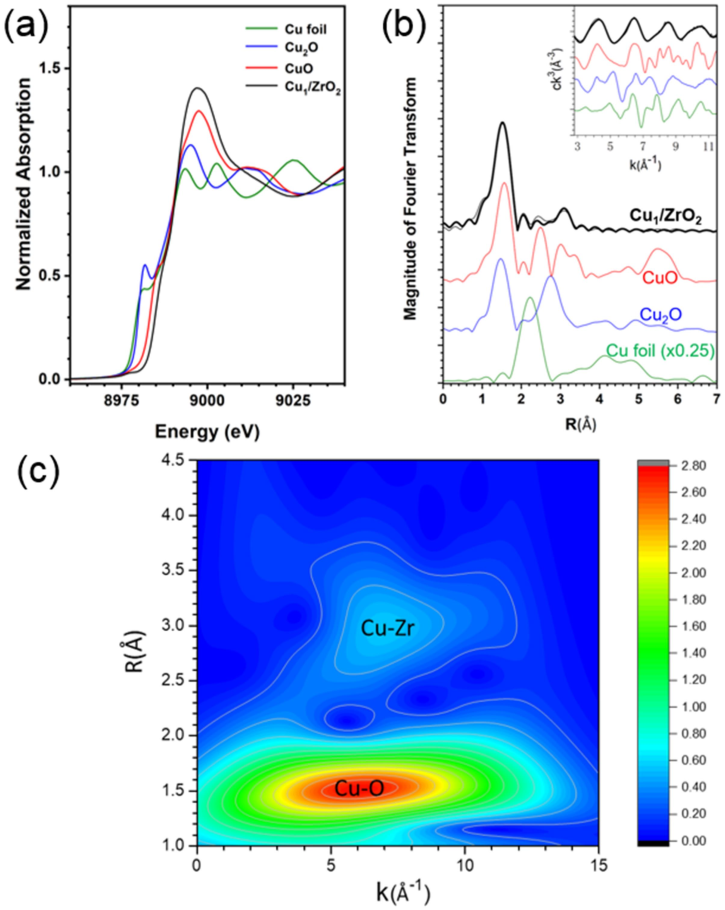

Figure S9. The XAS analysis of the Cu_1/ZrO_2 SAC prepared at 800 ºC: (a) Cu K-edge XANES spectra; (b) EXAFS curve and the fitting curves of Cu_1/ZrO_2 and reference samples at R and K space; (c) Wavelet transform of Cu_1/ZrO_2 SAC.

As shown in **Fig. S9a**, the edge position of Cu_1/ZrO_2 was slightly higher than that of CuO, which indicates that the valence state of the Cu species in the Cu_1/ZrO_2 SACs was about +2. The corresponding Fourier transform (FT) EXAFS spectra of Cu_1/TiO_2 and the references (Cu foil, CuO and Cu_2O) were illustrated in **Fig. S9b**. The Cu_1/ZrO_2 SACs exhibited an obvious FT peak located at ~1.94 Å, which could be mainly attributed to the scattering of Cu-O coordination. Moreover, there is no Cu-Cu shell peak in the sample, further suggesting that only atomically dispersed Cu atoms were present in the ZrO_2-based sample. In addition, the fitting curve in **Fig. S9b** revealed that the Cu−O coordination number in the Cu_1/ZrO_2 is 3.7 ± 0.3, along with the fitting parameters summarized in **Table S3**. The wavelet transform of Cu_1/ZrO_2 sample in **Fig. S9c** indicated the existence of Cu-O and Cu-Zr bonds within the Cu_1/ZrO_2 SAC sample. The XANES and EXFAS results of Cu_1/ZrO_2 SAC verified that Cu atoms were atomically dispersed on the ZrO_2 shell.

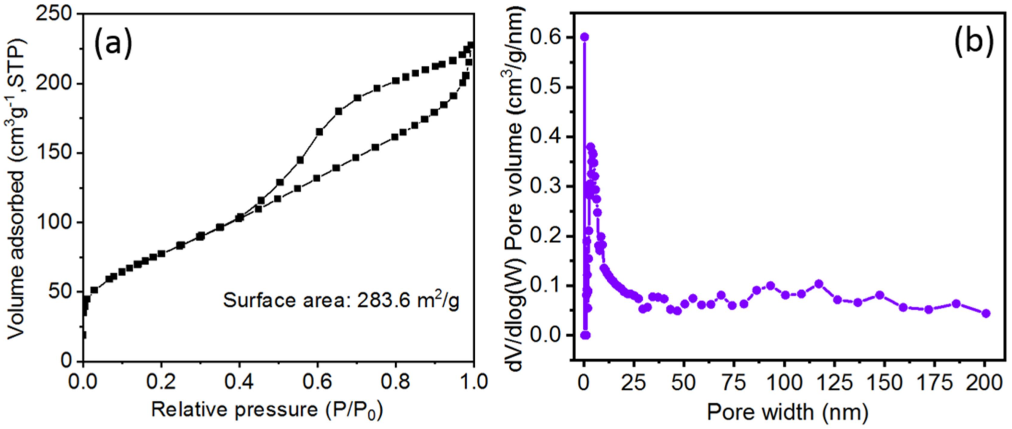

Figure S10. The N_2 adsorption-desorption isotherm of the Ni_1/TiO_2 SAC prepared at 800 °C (a) and pore size distribution (b).

As shown in ***Figure S10a***, the as-prepared Ni_1/TiO_2 SACs displayed a Type IV isotherm with a well-defined hysteresis loop which is related to mesopores. The BET surface area of the material is ~284 m^2/g. As observed from the pore size distribution in ***Figure10b***, the main pore size of Ni_1/TiO_2 SAC sample is about 4.6 nm.

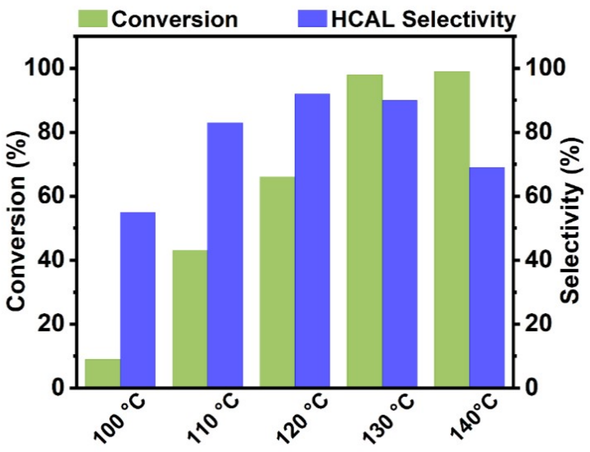

Figure S11. The catalytic performance of the Ni_1/TiO_2 SACs prepared at 800 °C toward the selective hydrogenation of cinnamaldehyde under different reaction temperatures.

The results show that 130 ºC is the optimal reaction temperature when both conversion and selectivity are taken into account.

Table S4. Selective hydrogenation results of the Ni_1/TiO_2 SACs prepared at 800 °C at different reaction temperatures.

Catalysts	Reaction temperature (ºC)	Conversion (%)	Selectivity (%)		
			**COL**	**HCAL**	**HCOL**
Ni_1/TiO_2 SACs	140	99	5	69	26
	**130**	**98**	**3**	**90**	**7**
	120	66	6	92	2
	110	43	17	83	-
	100	9	45	55	-

Reaction condition: 25 mg of catalyst, substrate (0.4 mmol), isopropanol (25 mL), H_2 (3 MPa), 2 h

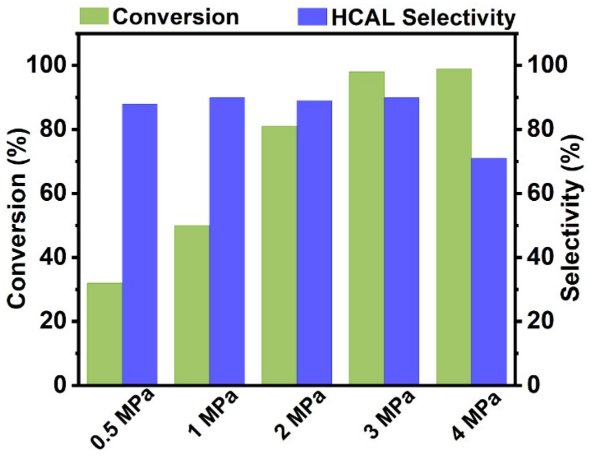

Figure S12. The catalytic performance of the Ni_1/TiO_2 SACs prepared at 800 °C toward the selective hydrogenation of cinnamaldehyde under the different H_2 pressure.

As shown in **Figure S12**, with the increase of H_2 pressure from 0.5 to 3 MPa, the conversion increased while the selectivity remained almost constant. Further increasing the H_2 pressure to 4 MPa, only a slight increment of the conversion was observed, but the selectivity dropped greatly. Thus, 3 MPa of the H_2 pressure was sufficient to achieve both high conversion and excellent selectivity.

Table S5. Selective hydrogenation results of the Ni_1/TiO_2 SACs prepared at 800 °C under different reaction pressures.

Catalysts	Reaction pressure (MPa)	Conversion (%)	Selectivity (%)		
			**COL**	**HCAL**	**HCOL**
Ni_1/TiO_2 SACs	4	99	6	71	23
	**3**	**98**	**3**	**90**	**7**
	2	81	4	89	7
	1	50	10	90	-
	0.5	32	12	88	-

Reaction condition: 25 mg of catalyst, substrate (0.4 mmol), isopropanol (25 mL), 2 h

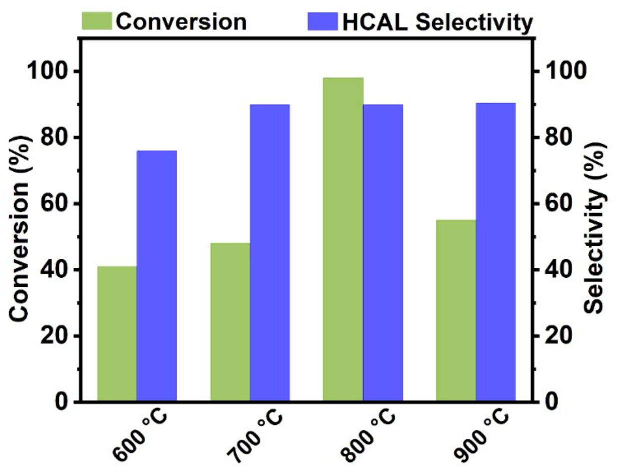

Figure S13. The catalytic performance of different Ni_1/TiO_2 SACs toward the selective hydrogenation of cinnamaldehyde, and the Ni_1/TiO_2 SACs were prepared at different calcination temperatures.

Table S6. Selective hydrogenation results of the Ni_1/TiO_2 SACs at different calcination temperatures.

Catalysts	Calcination temperature (ºC)	Conversion (%)	Selectivity (%)		
			**COL**	**HCAL**	**HCOL**
Ni_1/TiO_2 SACs	600	41	23	76	1
	700	48	10	90	-
	**800**	**98**	**3**	**90**	**7**
	900	80	4	94	2

Reaction condition: 25 mg of catalyst, substrate (0.4 mmol), isopropanol (25 mL), H_2 (3 MPa), 2 h

Table S7. Selective hydrogenation results of the TiO_2 catalysts loaded with different metals, prepared at 800 °C.

Catalysts	Conversion (%)	Selectivity (%)		
		**COL**	**HCAL**	**HCOL**
Fe_1/TiO_2	14	45	55	-
Co_1/TiO_2	23	27	73	-
**Ni_1/TiO_2**	**98**	**3**	**90**	**7**
Cu_1/TiO_2	16	47	51	2

Reaction condition: 25 mg of catalyst, substrate (0.4 mmol), isopropanol (25 mL), H_2 (3 MPa), 2 h

Table S8. Selective hydrogenation results of the TiO_2 catalyst prepared at 800 °C with different Ni loading amounts.

Catalysts	Conversion (%)	Selectivity (%)		
		**COL**	**HCAL**	**HCOL**
TiO_2	27	47	42	11
Ni_1/TiO_2 0.08 wt%	42.6	9	91	-
Ni_1/TiO_2 0.2 wt%	58.5	7	93	-
**Ni_1/TiO_2 0.4 wt%**	**98**	**3**	**90**	**7**
Ni/TiO_2 0.8 wt%	94	5	90	5

Reaction condition: 25 mg of catalyst, substrate (0.4 mmol), isopropanol (25 mL), H_2 (3 MPa), 2 h

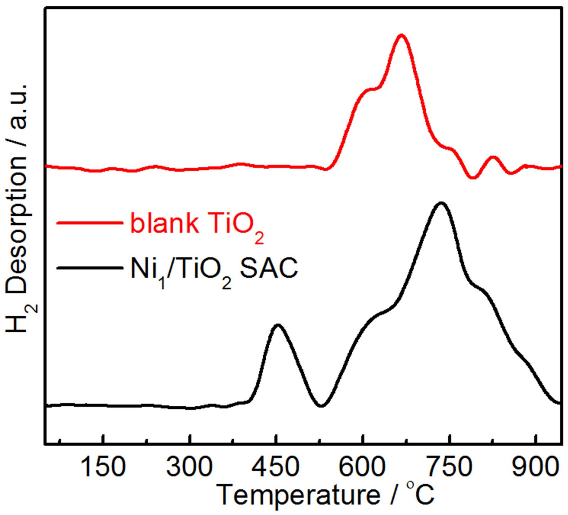

Figure S14. H_2 TPD profiles of blank TiO_2 and Ni_1/TiO_2 single atom catalyst

Hydrogen spillover is a widespread phenomenon in heterogeneous catalysis and is often used as the explanation for the catalytic phenomenon, which could be detected by H_2-TPD measurement. [1] Obviously, the area of the H_2-TPD peak in the range from 525 to 900 ºC in the case of the Ni_1/TiO_2 SAC was much larger than that in the case of blank TiO_2 support (**Figure S14**). This may result from the hydrogen spillover effect in the as-prepared Ni_1/TiO_2 SAC. The hydrogen molecules first adsorb and dissociate into hydrogen atoms at the sites of Ni single atoms, and then the hydrogen atoms move across the interfaces between Ni atoms and the TiO_2 support, and subsequently spread across the surface of the TiO_2 support. [1] Besides, a new H_2 desorption peak appeared at the lower temperature of 450 ºC when Ni single atoms were embedded within the TiO_2 support. The peak was assigned to desorption of H_2 from Ni atoms. [2] Moreover, the peak area of H_2-TPD could be related to the amount of active species. The more hydrogen adsorbed on the support, the higher the catalytic activity was obtained. [3] The improvement in the catalytic performance of Ni_1/TiO_2 SAC resulted from the enhancement in the amount of chemisorbed hydrogen (hydrogen spillover), which also indicated that there were the cooperative effects between the Ni single atoms and the TiO_2 support. Thus, the non-noble Ni single atoms were essential for the selective hydrogenation of cinnamaldehyde in the case of the Ni_1/TiO_2 SAC. The cooperative effect between the Ni species and the TiO_2 support guaranteed the excellent catalytic activity of Ni_1/TiO_2 SAC toward the selective hydrogenation of cinnamaldehyde.

Table S9. Selective hydrogenation results of different catalysts prepared at 800 °C.

Catalysts	Conversion (%)	Selectivity (%)		
		**COL**	**HCAL**	**HCOL**
Ni_{NPs}/ZrO_2	42	13	87	-
**Ni_{NPs}/TiO_2**	**80**	**10**	**82**	**8**
**Ni_1/TiO_2**	**98**	**3**	**90**	**7**
Ni_1/ZrO_2	87	8	85	7
Ni_{NPs}/SiO_2	50	22	70	8

Reaction condition: 25 mg of catalyst, substrate (0.4 mmol), isopropanol (25 mL), H_2 (3 MPa), 2 h

Table S10. The cycling performance of the Ni_1/TiO_2 SACs prepared at 800 °C.

Catalysts	Cycle number	Conversion (%)	Selectivity (%)		
			**COL**	**HCAL**	**HCOL**
	1	98	3	90	7
	2	94	7	84	9
Ni_1/TiO_2	3	93	6	92	2
	4	93	4	89	7
	5	89	3	91	4

Table S11. Selective hydrogenation results of ZrO_2 catalysts prepared at 800 °C loaded with different metals.

Catalysts	Conversion (%)	Selectivity (%)		
		**COL**	**HCAL**	**HCOL**
Fe_1/ZrO_2	78	41	29	30
Co_1/ZrO_2	19	22	78	-
**Ni_1/ZrO_2**	**87**	**8**	**85**	**7**
Cu_1/ZrO_2	60	28	49	23

Reaction condition: 25 mg of catalyst, substrate (0.4 mmol), isopropanol (25 mL), H_2 (3 MPa), 2 h

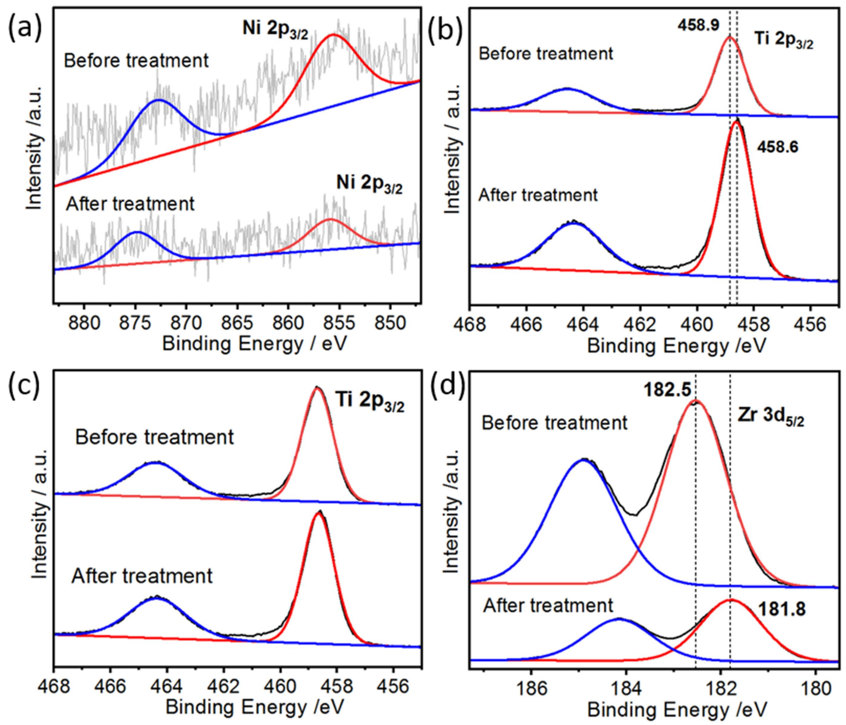

Figure S15. Ni2p (a) and Ti2p (b) spectra of Ni_1/TiO_2SAC; Ti2p spectra of the blank TiO_2 support (c) and Zr3d XPS spectra of the Ni_1/ZrO_2 SAC (d) before and after treatment under H_2 atmosphere (3 MPa) at 130 ºC for 2 h, measured by ex-situ XPS.

To explain the catalytic difference between Ni_1/TiO_2 and N_1/ZrO_2 SACs, ex-situ XPS measurement was conducted. The Ni_1/TiO_2 and N_1/ZrO_2 SACs were pretreated in H_2 atmosphere (3 MPa) at 130 ºC for 2 hours before the *ex-situ* XPS measurement. The experimental parameters of the pretreatment were the same as those used for the hydrogenation of cinnamaldehyde. As shown in **Figure S15a**, no obvious difference in the binding energy for Ni $2p_{3/2}$ was observed after the treatment. Thus, the valence state of Ni species remained approximately +2 even after the treatment, which indicated that the catalytic activity of the Ni_1/TiO_2 SACs exhibited in the selective hydrogenation process originated from the Ni species with delta close to +2 rather than those with delta approximately being 0.[4] The results were in good agreement with those observed by XANES and EXAFS (**Figure 3**).

Meanwhile, after the treatment, the binding energy of Ti $2p_{3/2}$ in Ni_1/TiO_2 SAC shifted from 458.9 to 458.6 eV (**Figure S15b**). However, the binding energy of Ti $2p_{3/2}$ in blank TiO_2 remained unchanged after being treated under the same conditions (**Figure S15c**). The difference could be ascribed to the hydrogen spillover. The embedding of Ni atoms within the TiO_2 support could promote the reduction of the Ti species in the TiO_2 support, which also provided evidence for the interaction between the Ni atom and the TiO_2 support. As shown in

Figure S15d, in the case of the Ni_1/ZrO_2 SAC, the binding energy of Zr $3d_{5/2}$ greatly decreased from 182.5 eV to 181.7 eV after the treatment under the same conditions.[5] The shift of the binding energy of Zr $3d_{5/2}$ was remarkably larger than that of Ti $2p_{3/2}$ in the Ni_1/TiO_2 SAC. Thus, the interaction between the Ni atom and the ZrO_2 support was different from that between Ni atom and the TiO_2 support. Ni atoms influenced the TiO_2 and ZrO_2 supports to different extent. The low-energy shift of binding energy for Ti species or Zr species in oxides indicated the loss of oxygen atoms in the oxide supports during the treatment. The loss of oxygen would alter the coordination environments of Ni atoms, and further affect the adsorption properties as well as the catalytic performance of Ni sites. [3] The low-shift of the binding energy of Zr $3d_{5/2}$ was remarkably larger than that of Ti $2p_{3/2}$ during the hydrogenation process, which may contribute to the different catalytic performance between the Ni_1/TiO_2 and Ni_1/ZrO_2 SACs.

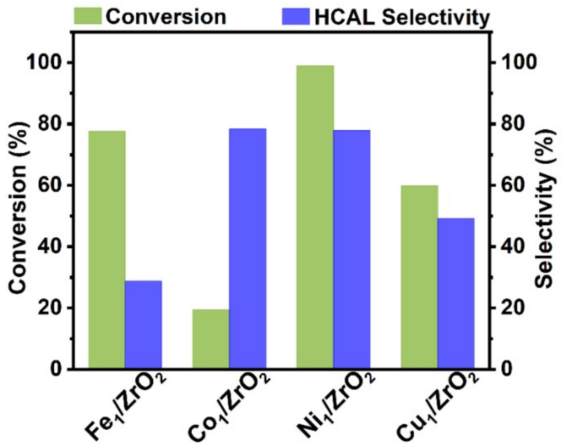

Figure S16. The catalytic performance of different ZrO_2-based SACs prepared at 800 °C toward the selective hydrogenation of cinnamaldehyde, including Fe_1/ZrO_2, Co_1/ZrO_2, Ni_1/ZrO_2, and Cu_1/ZrO_2.

Table S12. Selective hydrogenation results of the blank ZrO_2 and Ni_1/ZrO_2 catalysts prepared at different calcination temperatures.

Catalysts	Calcination temperature (ºC)	Conversion (%)	Selectivity (%)		
			**COL**	**HCAL**	**HCOL**
Ni_1/ZrO_2	600	29	31	65	4
	700	58	17	76	7
	**800**	**87**	**8**	**85**	**7**
	900	43	50	43	7
ZrO_2	800	12	16	79	5

Reaction condition: 25 mg of catalyst, substrate (0.4 mmol), isopropanol (25 mL), H_2 (3 MPa), 2 h

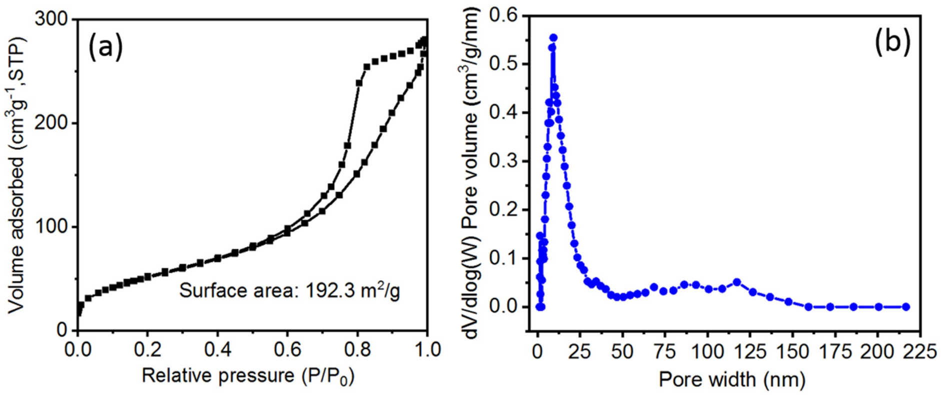

Figure S17. The N_2 adsorption-desorption isotherm of the Ni_1/TiO_2 sample prepared at 900 °C (a) and pore size distribution (b).

Table S13. Summary of different heterogeneous catalysts for the selective hydrogenation of cinnamaldehyde in recently published works.

Catalysts	Reaction conditions		Conv. /%	HCAL /%	COL /%	TOF /h^{-1}	Ref.
	T/ ºC	P/ MPa					
Ni_1/TiO_2	130	3	98	90	3	102	This work
$NiIr/TiO_2$	80	2	97.8	95.4	N.A.	93.4	6
Ni-in-ANTs	80	2	99	91	N.A.	1512	7
Ni/TiO_2-DP	120	2	70	64.6	29.9	2.52	8
$mSiO_2@Ni/SiO_2@mSiO_2$	80	3.38	100	100	N.A.	23.8	9
$Pt_3Ni@Ni_{32}Cu(OH)_2$-2 NWs [a]	50	3	98.5	87.9	4.5	72	10,11
Pd/BP	25	0.1	100	92	N.A.	50.4	12
$Au-PVP/SiO_2$	150	2	6.4	60	30	125	13
$Au-PVP/SiO_2$-12UVO	150	2	18	87	9	352	13
MIL-101(Cr)@Pt@MIL-101(Fe)[2.9]	25	3	99.8	0.8	95.6	16.9	14
MIL-101(Fe)@Pt	25	3	45	9.6	86.4	122.1	14
SPhF-Ni_2P	80	1	100	0	98.8	87	15
Porous $PtNi_{2.20}$ NWs@Ni/Fe_4-MOF	40	3	100	1.65	83.3	77.6	16
C10F-$Fe_{0.33}Pt_{0.67}$	50	0.1	71.1	1.5	94	87	17
Au clusters coated with tert-butyl(naphthalene-1-yl)phosphine oxide [b]	60	4	> 99	N.A.	> 99	2.8	18,19

[a] the catalyst is from ref. 10, and the TOF value is calculated by ref. 11 (Table 2); [b] the catalyst is from ref. 18, and the TOF value is calculated by ref. 19 (Table S4).

References:

[1] M. Xiong, Z. Gao, Y. Qin, *ACS Catal.* **2021**, *11*, 3159.

[2] Y. Guo, S. Mei, K. Yuan, D. J. Wang, H. C. Liu, C. H. Yan, Y. W. Zhang, *ACS Catal.* **2018**, *8*, 6203.

[3] J. Zhang, Z. Gao, S. Wang, G. Wang, X. Gao, B. Zhang, S. Xing, S. Zhao, Y. Qin, *Nat. Commun.* **2019**, *10*, 4166.

[4] W. Zang, T. Sun, T. Yang, S. Xi, M. Waqar, Z. Kou, Z. Lyu, Y. P. Feng, J. Wang, S. J. Pennycook, *Adv. Mater.* **2021**, *33*, 2003846.

[5] Y. Wang, L. Yao, Y. Wang, S. Wang, Q. Zhao, D. Mao, C. Hu, *ACS Catal.* **2018**, *8*, 6495.

[6] W. Lin, H. Cheng, L. He, Y. Yu, F. Zhao, *J. Catal.* **2013**, *303*, 110.

[7] Z. Gao, M. Dong, G. Wang, P. Sheng, Z. Wu, H. Yang, B. Zhang, G. Wang, J. Wang, Y. Qin, *Angew. Chem.* **2015**, *54*, 9006.

[8] M. G. Prakash, R. Mahalakshmy, K. R. Krishnamurthy, B. Viswanathan, *Catal. Sci. Technol.* **2015**, *5*, 3313.

[9] B. Li, H. C. Zeng, *ACS Appl. Mater. Interfaces* **2018**, *10*, 29435.

[10] P. Wang, Q. Shao, X. Cui, X. Zhu, X. Huang, *Adv. Funct. Mater.* **2018**, *28*, 1705918.

[11] X. Wang, X. Liang, P. Geng, Q. Li, *ACS Catal.* **2020**, 10, 2395.

[12] S. Fujiwara, N. Takanashi, R. Nishiyabu, Y. Kubo, *Green Chem.* **2014**, *16*, 3230.

[13] R. Y. Zhong, K. Q. Sun, Y. C. Hong, B. Q. Xu, *ACS Catal.* **2014**, *4*, 3982.

[14] M. Zhao, K. Yuan, Y. Wang, G. Li, J. Guo, L. Gu, W. Hu, H. Zhao, Z. Tang, *Nature* **2016**, *539*, 76.

[15] R. Gao, L. Pan, H. Wang, Y. Yao, X. Zhang, L. Wang, J. J. Zou, *Adv. Sci.* **2019**, *6*, 1900054.

[16] N. Zhang, Q. Shao, P. Wang, X. Zhu, X. Huang, *Small* **2018**, *14*, 1704318.

[17] K. B. Vu, K. V. Bukhryakov, D. H. Anjum, V. O. Rodionov, *ACS Catal.* **2015**, *5*, 2529.

[18] I. Cano, M. A. Huertos, A. M. Chapman, G. Buntkowsky, T. Gutmann, P. B. Groszewicz, P. W. N. M. Van Leeuwen, *J. Am. Chem. Soc.* **2015**, *137*, 7718.

[19] K. Yuan, T. Song, D. Wang, X. Zhang, X. Gao, Y. Zou, H. Dong, Z. Tang, W. Hu, *Angew. Chem. Int. Ed.* **2018**, *130*, 5810.
